# Bilateral spontaneous pneumothorax in critically-ill COVID-19 infants: About two cases

**DOI:** 10.1016/j.amsu.2021.103172

**Published:** 2021-12-06

**Authors:** Ilyass Laaribi, Hamza Mimouni, Zakaria Bouayed, Ghizlane El Aidouni, Samia Berrichi, Choukri Bahouh, Houssam Bkiyar, Brahim Housni

**Affiliations:** a Anesthesia, Intensive Care and Resuscitation Department, MOHAMMED VI University Hospital Center, Oujda, Morocco; b Faculty of Medicine and Pharmacy, Mohammed First University, Oujda, Morocco; c Simulation Center, Faculty of Medicine and Pharmacy, Oujda, Morocco

**Keywords:** COVID-19, Infant patients, Spontaneous pneumothorax

## Abstract

**Introduction:**

COVID-19 is an emerging infection, it is the first large-scale pandemic of the 21st century. Several complications have been described during this infection but spontaneous pneumothorax remains an uncommon complication, even more so in infants.

**Clinical presentation:**

We report two cases of a 9-month-old and 18-month-old males admitted to our department for the management of an acute respiratory distress due to a COVID-19 infection associated to a spontaneous pneumothorax successfully drained.

While one patient had a favorable outcome, the other was readmitted to our department for the management of a septic shock secondary to a urinary tract infection with a deadly outcome.

**Discussion:**

In this paragraph we describe known causes behind spontaneous pneumothorax, before detailing the different pathogenesis hypotheses linking pneumothorax to COVID-19, all while comparing data to the literature related to the adult population.

**Conclusion:**

Spontaneous pneumothorax is a serious complication associated with severe COVID-19 that can occur in infants and must be considered in the event of a respiratory aggravation or a persistent hypoxia.

## Highlights

- The full scope of COVID-19's complications is widening with every related work reported in the literature.
- Spontaneous pneumothorax in infants infected with COVID-19 is an unprecedented complication.
- Multiple hypotheses were proposed to explain the occurrence of a spontaneous pneumothorax but none have been conclusively proven

## 1 Introduction

COVID-19 is an emerging infection, it is the first large-scale pandemic of the 21st century, since its appearance in Wuhan in December 2019 [1], it has raised substantial concerns fueled by the disease's rapid progression throughout the World warranting its designation as a Public Health Emergency of International Concern by the World's Health Organization on January 2021. To this day according the official the WHO's official COVID-19 dashboard there have been 263 563 622 confirmed cases of COVID-19, including 5 232 562 deaths to this day [2]. Thousands of cases of infected children have been reported. However, the severity and the mortality rate remain low compared to the adult population [3].

Spontaneous pneumothorax is a rare complication of COVID-19 in adults, as only few cases have been reported [4,5]. This complication is even rarer in children. To this day, no cases of spontaneous pneumothorax in infants have been reported, we present 2 cases of infants with a spontaneous pneumothorax associated with a COVID-19 infection.

## 2 Clinical presentation

### 2.1 Case N ° 1

A 9-month-old male was admitted to the pediatric emergency department for a respiratory distress evolving for 5 days associated to a fever. His mother had tested positive for COVID-19 a week earlier. On admission the infant was conscious, hypotonic, febrile at 39 °C, tachycardic at 185 bpm, and tachypneic at 77 cpm. His oxygen saturation was 70% on ambient and 88% under 8L/min of oxygen. A decrease of the vesicular murmurs was noted bilaterally along with signs of respiratory distress.

**Fig. 1:**
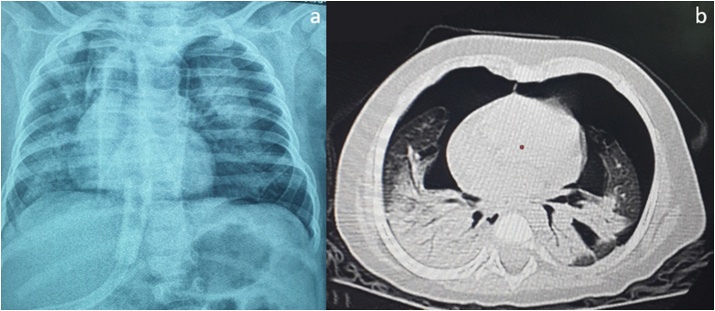
a. CXR showing left pneumothorax with foci of condensation. b. Axial sequence of a lung window chest-CT revealing a bilateral large pneumothorax with an interstitial alveolar pneumonitis and posterior consolidation.

The patient had a history of epilepsy under treatment since the age of 4 months-old. CXR showed a left pneumothorax with foci of condensation (Fig. 1a), a chest-CT was carried out revealing a large pneumothorax bilaterally, and a bilateral interstitial alveolar pneumonitis with posterior consolidation (Fig. 1b).

Laboratory tests showed a white blood count (WBC) of 7020/mm^3^(a 600/mm^3^ lymphocytes), D-dimers at 1.22 mg/L, C reactive protein (CRP) elevated at 111 mg/L, Lactate Dehydrogenase (LDH) at 1718 U/L and ferritin level at 2000 μg/L, interleukin 6 at 138 pg/ml. A SARS-CoV-2 RT-PCR test performed on a nasopharyngeal swab the next day came back positive.

The pneumothorax was drained using a bilateral chest tubes each connected to a single-bottle system, medical treatment consisted of dexamethasone 0,15 mg/kg/day and Amoxicillin/Clavulanic Acid 80mg/kg/day.

The regression and ultimately the disappearance of the pneumothorax was assessed using pleural ultrasonography, the drains were removed after 5 days, during which the patient was gradually weaned off oxygen and discharged within the 8th day of hospital stay.

### 2.2 Case N ° 2

An 18-month-old male infant, with no medical history other than a circumcision 2 weeks prior, with no notion of COVID-19 infection in the entourage, was admitted to the pediatric emergency department for a respiratory distress. The mother reported a very severe dry cough evolving for 2 days and a fever persistent despite symptomatic treatment. On admission, the infant was cyanotic, hypotonic, and tachypneic at 85 cpm, with intercostal, suprasternal indrawing and thoraco-abdominal rocking, his arterial oxygen saturation was 65% on ambient air and 87% under 10L/min of oxygen.

Complete blood count showed elevated WBC (15800/mm^3^) with lymphopenia 570/mm^3^; elevation of C-reactive protein (220 mg/L), Ferritin level (1052 μ/L), LDH (1612 U/L) and US troponin (183 ng/mL) with D-dimers at 3.15 mg/L. SARS-Cov-2 RT-PCR was performed coming back positive.

**Fig. 2:**
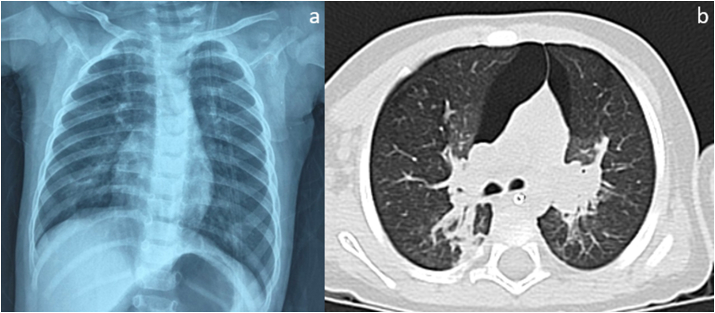
a. CXR showing no abnormalities. b. Axial sequence of a lung window chest-CT revealing a bilateral large pneumothorax with an interstitial alveolar pneumonitis.

CXR did not reveal any abnormalities (Fig. 2a). Chest-CT revealed an alveolar-interstitial pneumopathy along with a large bilateral anterior pneumothorax (Fig. 2b) which was drained promptly also using bilateral chest tubes each connected to a single-bottle system.

Medical treatment consisted of dexamethasone 0.15mg/kg/day and Amoxicillin/Clavulanic Acid 80mg/kg/day. The infant drains were removed three days later. The patient was transferred to pediatrics on the following day, however, on the 7th day of hospitalization he developed a urinary tract infection complicated by a refractory septic shock warranting a re-admission to our department, but died shortly thereafter.

## 3 Discussion

Spontaneous pneumothorax can be secondary to an underlying pulmonary pathology such as chronic obstructive pulmonary disease with emphysema, cystic fibrosis, tuberculosis, lung cancer, pneumocystis associated with HIV jiroveci pneumonia (PJP) or other cystic pneumonitis, it can also be a primary pneumothorax in the absence of any pulmonary disease [6,7], which was the case in our 2 patients.

Unlike the number of pneumothorax cases complicating COVID-19 in adults reported to the literature [8,9], no cases have been reported in infants.

No causal link has been proven between the occurrence of pneumothorax during COVID-19 to this day and its pathogenicity is still yet to be established. There are several possible hypotheses, such as the presence of pneumatoceles or cysts in COVID-19 patients in the absence of any mechanical ventilation, two cases have been reported of patients with cystic lung lesions linked to COVID-19, one of which was complicated by a pneumothorax [10]. Which is similar to cases of spontaneous pneumothorax in HIV patients and PJP pneumonia [11]. Another case describes a patient with no underlying lung pathology who developed a giant emphysematous bulla during COVID-19 pneumonia, which ended up bursting and causing a pneumothorax [5]. Moreover, none of the cases described were under mechanical ventilation, which suggests that the barotrauma associated with positive pressure ventilation cannot explain the formation of cysts that may subsequently lead to a secondary pneumothorax [4,10]. In our case, none of the 2 infants underwent mechanical ventilation, which a barotrauma as a cause of the pneumothorax.

Another hypothesis incriminates prolonged coughing as a possible trigger, which is a common symptom of COVID-19 [12,13], and could increase intra-thoracic pressure leading to a pneumothorax in the presence of alveolar damage due to COVID-19 pneumonia-related inflammation or ischemic parenchymal injury [14,15]. This mechanism could explain the pneumothorax in our second case.

In our case, the 2 infants presented lymphopenia, elevated reactive protein C, LDH, ferritin and D-dimers levels, as well as interleukin 6 (Table 1). These inflammation biomarkers suggest a significant inflammatory state and therefore a more severe pulmonary damage [16].

**Table 1:** Laboratory results of the 2 cases.

	GB	Lymphocytes	CRP	Ferritinemia	D-dimer	Troponin	LDH	IT 6
Case 1	7020/mm^3^	600/mm^3^	111 mg/L	2000 μ/L	1.22 mg/L	96 ng/ml	1718 U/L	138 pg/ml
Case 2	15800/mm^3^	570/mm^3^	220mg/L	1052 μ/L	3.15 mg/L	183ng/ml	1612 U/L	101pg/ml

Indeed, recently published studies on the mechanisms of pulmonary lesions induced by COVID-19 assume that the hyperactivity and deregulation of the immune response during the cytokine storm is responsible for more severe lung injuries [17]. In fact a high inflammatory state in COVID-19 adult patients suffering from a primary pneumothorax has been reported to the literature [18].

The occurrence of a pneumothorax in COVID-19 patients has been considered a marker of grave prognosis [9,19]. However, none of our patients died from their respiratory injury. Our case series therefore aligns with the largest multicenter case series published to date on pneumothorax in COVID-19 [8] among other studies.

## 4 Conclusion

Spontaneous pneumothorax is a rare complication of COVID-19. It can occur at any time during the disease.

As information around COVID-19 in children continues to evolve, we highlight an important life-threatening complication associated with severe COVID-19 in infancy. Our case report is particularly interesting because it draws attention to the importance of considering a pneumothorax in infants in front of a respiratory aggravation or a persistent hypoxia even in the absence of mechanical ventilation.

This work is reported in line with the 2020 SCARE guidelines [20].

## Patient consent

Written informed consent was obtained from the parents (given that our patients are minors) for publication of this case report and accompanying images. A copy of the written consent is available for review by the Editor-in-Chief of this journal on request.

## Provenance and peer review

Not commissioned, externally peer reviewed.

## Please state any conflicts of interest

We have no conflicts of interest.

## Please state any sources of funding for your research

This work hasn't received any funding.

## Ethical approval

This is a case report, therefore Ethics committee/IRB approval is not required.

## Consent

Written informed consent was obtained from the parents (given that our patients are minors) for publication of this case report and accompanying images. A copy of the written consent is available for review by the Editor-in-Chief of this journal on request.

## Author contribution

ILYASS LAARIBI: study conception, data collection and analysis, writing and editing. HAMZA MIMOUNI: data collection and analysis. ZAKARIA BOUAYED: data analysis and writing. GHIZLANE EL AIDOUNI: contributor. SAMIA BERRICHI: contributor. CHOUKRI BAHOUH: contributor. HOUSSAM BKIYAR: Supervision and review data validation. HOUSNI BRAHIM: Supervision and review data validation.

## Registration of research studies

1. Name of the registry:
2. Unique Identifying number or registration ID:
3. Hyperlink to your specific registration (must be publicly accessible and will be checked):

## Guarantor

ILYASS LAARIBI.
